# Robot-assisted implantation of additively manufactured patient-specific orthopaedic implants: evaluation in a sheep model

**DOI:** 10.1007/s11548-023-02848-8

**Published:** 2023-03-01

**Authors:** Tom Williamson, Stewart Ryan, Ulrich Buehner, Zac Sweeney, Dave Hill, Bill Lozanovski, Endri Kastrati, Arman Namvar, Thierry Beths, Darpan Shidid, Romane Blanchard, Kate Fox, Martin Leary, Peter Choong, Milan Brandt

**Affiliations:** 1 RMIT Centre for Additive Manufacturing, RMIT University, Melbourne, Australia; 2 Translational Research and Animal Clinical Trial Study Group (TRACTS), Faculty of Veterinary and Agricultural Sciences, University of Melbourne, Melbourne, Australia; 3 Stryker, Sydney, Australia; 4 Department of Surgery, University of Melbourne, Melbourne, Australia; 5 Orthopaedic Department, St Vincent’s Hospital, Melbourne, Australia

**Keywords:** Bone tumours, Surgical robotics, Patient-specific implants, Orthopaedic oncology, Robotic surgery

## Abstract

**Purpose:**

Bone tumours must be surgically excised in one piece with a margin of healthy tissue. The unique nature of each bone tumour case is well suited to the use of patient-specific implants, with additive manufacturing allowing production of highly complex geometries. This work represents the first assessment of the combination of surgical robotics and patient-specific additively manufactured implants.

**Methods:**

The development and evaluation of a robotic system for bone tumour excision, capable of milling complex osteotomy paths, is described. The developed system was evaluated as part of an animal trial on 24 adult male sheep, in which robotic bone excision of the distal femur was followed by placement of patient-specific implants with operative time evaluated. Assessment of implant placement accuracy was completed based on post-operative CT scans.

**Results:**

A mean overall implant position error of 1.05 ± 0.53 mm was achieved, in combination with a mean orientation error of 2.38 ± 0.98°. A mean procedure time (from access to implantation, excluding opening and closing) of 89.3 ± 25.25 min was observed, with recorded surgical time between 58 and 133 min, with this approximately evenly divided between robotic (43.9 ± 15.32) and implant-based (45.4 ± 18.97) tasks.

**Conclusions:**

This work demonstrates the ability for robotics to achieve repeatable and precise removal of complex bone volumes of the type that would allow en bloc removal of a bone tumour. These robotically created volumes can be precisely filled with additively manufactured patient-specific implants, with minimal gap between cut surface and implant interface.

## Introduction

Local control of bone tumours involves surgical excision of tumour tissue in one piece (en bloc), including a margin of non-diseased bone. Bone reconstruction after tumour removal may be achieved through biological approaches (auto- or allografts) or the use of modular prostheses [1]. As bone sarcomas are relatively rare and may present in a range of locations with a variety of shapes [2], they are well suited to the use patient-specific implants [3]. Additive manufacturing allows for the creation of highly complex implant geometries that closely match a patient’s anatomy, as well as the creation of lattice and porous structures and tailoring of implant mechanical properties [4].

Each of these reconstruction approaches relies on the surgeon’s ability to accurately remove bone. A previous study by Cartiaux et al. [5] demonstrated that it is difficult for even experienced surgeons to consistently achieve accurate cuts when removing tumours. Accurate bone removal is critical to the success of a limb-sparing procedure: variations from surgical plan may result in positive surgical margins, associated with a higher probability of local tumour recurrence and poorer outcomes [6]. Lower accuracy may also result in wider margins to account for cutting errors and potentially the removal of excess bone or other anatomical structures [7]. The relative locations of cuts determine how the implant fits into the created space. Gaps between cut bone and implant interface have been shown to reduce the effectiveness of the bone ingrowth process, and poor initial implant fit may decrease the longevity of the implant [8]. Gaps as small as 1 mm have been shown to adversely affect short-term implant stability and bone ingrowth [9].

Assistive technology is commonly used in bone tumour excision: surgical navigation was first applied in this context in 2004 [10] and has been widely used since [11–13]. The use of patient-specific instruments (PSI) has also been extensively described [14–16]. Comparisons between navigation and PSI by Bosma et al. [17] and Wong et al. [18] have made that both approaches improve resection accuracy. Both PSI [19] and navigation [20] have been shown to reduce overall recurrence rates when compared to manual surgery.

Surgical robotics has seen a significant increase in adoption over the last decade [21]. In orthopaedics, robots are primarily used to assist with the placement of off-the-shelf implants [22], and there are limited examples of work completed on the use of robotics for bone tumour excision. Khan et al. demonstrated improved accuracy when using a haptic-guided robotic system for planar cuts as compared to manual surgery [23]. Kong et al. [24] described the development and initial evaluation of a prototype robotic system for bone tumour removal, performing straight and circular cuts in animal cadaver tissue. Existing technologies largely limit surgeons to combinations of planar cuts. However, robotics may allow performance of more complex geometries conforming closely to a tumour shape, saving bone and other anatomical structures [25].

This work was performed as part of a 5-year project investigating the potential for improved patient outcomes in bone tumour resection through a combination of patient-specific implants and surgical robotics. Below, we describe the development and evaluation of a robotic system for bone tumour excision, evaluating the performance of complex osteotomy geometries and characterizing the achievable accuracy in an animal trial. We hypothesized that:

1. Robotics allows the consistent and accurate creation of complex cutting geometries simulating en bloc removal of bone tumours.
2. Created bone defects can be precisely filled with patient-specific additively manufactured implants manufactured a priori, with minimal gap between cut surface and implant.

## Materials and methods

The focus of this work was the assessment of a robot-assisted approach for placement of patient-specific implants, evaluating the achievable accuracy, workflow and intraoperative time. This was performed as part of an animal trial designed to compare bone ingrowth and biomechanical properties of solid and lattice patient-specific implants in combination with surgical robotics. In-growth and biomechanical outcomes will be described in subsequent publications; however, the individual cohorts are relevant to this work in relation to the timings of post-operative CT scans. Descriptions of the implant design and manufacturing process have been previously documented [4, 26].

### Overall animal study design

A total of 24 healthy adult Merino wethers were used in the trial. All animals were treated according to requirements for animal wellbeing and welfare, with the study approved by the University of Melbourne animal ethics committee [Ethics ID: 2021-10442-14222-5]. Sheep were randomly assigned an ID and allocated to an implant type (lattice or solid) and survival period. The study was separated into three phases:

- Two sheep were utilized for a pilot evaluation ensuring procedure safety and effectiveness. These were euthanized after 12 weeks and formed part of the histology cohort.
- An additional 8 sheep were designated as part of the 12-week survival period (histology) cohort.
- The remaining 14 sheep formed the biomechanical cohort, with a post-surgery survival period of 24 weeks.

All sheep underwent pre-operative CT of both femurs, surgical intervention and 12-week post-operative CT. CT scans were performed under general anaesthesia using a clinical scanner (Siemens Somatom Emotion 16, Siemens Healthineers, Germany). Additional interventions were performed depending on cohort as outlined in Table 1. Permission was given by the ethics committee to perform only one post-operative CT: the timing of these was defined to allow the maximum amount of information to be obtained about bone ingrowth and remodelling.

**Table 1:** Final animal study cohorts, survival periods and performed interventions relevant to this work. The overall study design effects this work only in the timing of post-operative CT scans, and that four procedures were performed posthumously due to the termination of the trial after 20 surgeries

Cohort	No. sheep	Survival period	Relevant interventions
Histology	10	12 weeks	Pre-op CT, surgery, 8 week CT, 12 week post-euthanasia CT
Biomechanical	10	24 weeks	Pre-op CT, surgery, 12 week CT, 24 week post-euthanasia CT
Biomechanical (t0)	4	0 weeks	Pre-op CT, surgery, immediate post-surgery CT

Three sheep were euthanized before the specified time point due to fractures of the operative leg occurring 1 day to 1 week after surgery. After the third fracture, the trial was stopped and the four remaining unoperated sheep euthanized at the request of the ethics committee. These four sheep were operated posthumously, providing additional accuracy data and time zero biomechanical testing data.

### Surgical planning and implant design

A custom surgical planning and implant design software was developed based on the open-source platform MITK (Medical Image Interaction Tool Kit, DKFZ, Germany), allowing segmentation of structures, definition of cuts, robotic path generation and implant design.

**Fig. 1:**
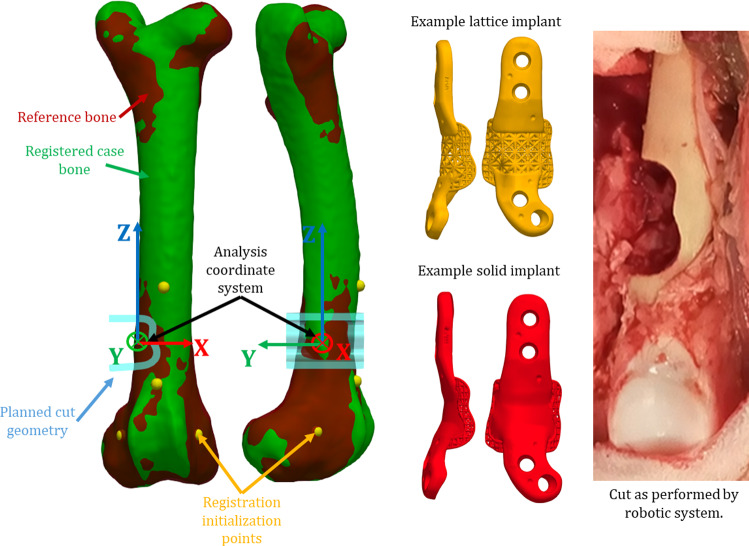
Sheep 4 femur (green) registered to reference bone (dark red) and reference cutting geometry (pale blue). Approximate locations of registration initialization points (yellow spheres) and overall analysis coordinate system are also shown. Examples of the two implant types (lattice, upper in yellow and solid with ingrowth layer, lower in red) are shown in the centre. An intraoperative craniocaudal view of the performed cut is shown on the right

The defined cut geometry and location was the same for all sheep. A “reference” plan was generated based on an average sheep bone geometry generated from pre-trial sheep femur data. A curved planar conformal cutting geometry was defined by manually placing points on the bone surface, with a smooth cutting path generated by interpolation. The cut was designed such that it would be difficult for a surgeon to reproduce without robotic assistance and based on preliminary tumour shape analysis. The cut was located on the lateral side of the right femur towards the distal end: the lateral position was chosen in consultation with the ethics committee to minimize potential post-surgery discomfort. The distal position was selected to include regions of cortical and trabecular bone, while mimicking common tumour locations. The reference plan is shown in Fig. 1.

Implants were designed taking into consideration expected loading and manufactured on a commercial laser bed powder fusion printer (SLM125, SLM Solutions, Germany) from Ti6Al4V ELI powder (SLM Solutions, Germany). All samples were deburred, cleaned via dry-ice blasting and repeated ultrasonic cleaning cycles, and autoclave sterilized before implantation.

Each patient-specific cutting plan was generated as follows:

- Pre-operative CT was imported into the planning software and the right femur manually segmented.
- The segmented bone was registered to the reference plan by manual alignment followed by iterative closest point (ICP) matching. The computed transformation was applied to the image data, aligning it with the reference plan.
- Robotic cutting paths and velocities were generated based on the reference cut geometry, aligned bone geometry and bone density extracted from aligned CT data.
- Four registration initialization points were defined at the medial and lateral condyles, patellar groove and proximally on the femur shaft.
- The bone geometry, robot path and registration points were exported as a custom surgical plan file.

### Robotic system for bone tumour excision

The prototype surgical robot consisted of a Denso VS-087 (Denso, Japan) arm fixed to a wheeled base (Rhino Cart, Stronghand Tools, USA), modified with prototype joint motor controllers and control software. A custom end-effector was developed allowing fixation of a Stryker Pi-Drive motor (Pi-Drive + , Stryker USA) and handpiece (PD Series Straight M, Stryker, USA), as well as a force-torque (FT) sensor (Mini-45, ATI, USA) and an off-the-shelf stainless-steel router (5120-071-223s1, Stryker, USA), at the robot wrist. The wrist interface was designed such that existing Stryker MAKO surgical drapes could be utilized. Control of the cutting motor and irrigation was performed through a Stryker Core 2 console (Stryker, USA).

**Fig. 2:**
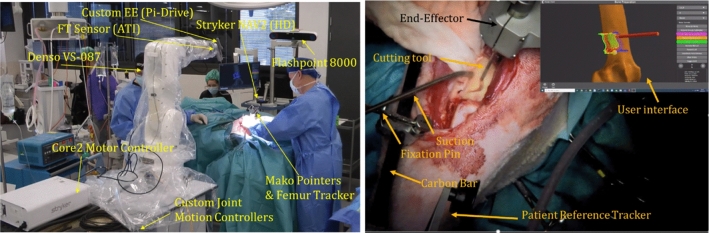
Left: developed system in the operating room. Right: surgical site during supervised autonomous bone cutting and user interface with overview of current robot and procedure state

An obsolete Stryker NAV-II system was modified with high-definition monitors, an updated PC and a prototype tracking system (Flashpoint 8000, Stryker, USA). A custom interface software was developed allowing patient-to-image registration and communication with the robot, tracking system, cutting motor and irrigation. MAKO tracking markers and registration tools were utilized for registration and patient tracking. The developed system is shown in Fig. 2.

### Surgical approach

Sheep were anaesthetized and the right-hind leg shorn and prepared with chlorhexidine and alcohol. The sheep was moved onto the operating table where it was placed in left lateral recumbency with the right leg upward. Draping was performed and an approximately 10-cm-long incision made from the mid-femoral shaft to the distal end of the femur on the lateral side of the right leg. The quadriceps muscles were retracted and the patella medially luxated. Once access was achieved, two positive profile pins were placed into the femur at the lateral condyle and percutaneously at the greater trochanter. A carbon-fibre external fixation rod and stainless-steel fixation clamps (SK ESF series, IMEX Veterinary, USA) were attached to the pins. The patient reference tracker (Mako Femoral Tracker, Stryker, USA) was attached to the fixation rod along with an additional carbon bar to allow fixation of the apparatus to the side rail of the operating table. The sheep was then rolled into dorsal recumbency and patient-to-image registration performed. During mobilization of the sheep, care was taken that sterile areas were touched only by scrubbed personnel and did not come into contact with non-sterile equipment.

After registration, the fixation hardware was attached to the operating table side rail and the robot moved to the operating position. The end-effector was moved into position by the surgeon using admittance control. Once within 20 mm of the cut path the robot switched to supervised automated cutting mode and completed the pre-planned cut. Cutting parameters used were:

- Cutting depth of 2 mm per pass,
- Tool rotational speed of 13′000 RPM,
- Variable feedforward based on tissue density information extracted from CT:
  - 5 mm/sec when moving through low-density areas (air, marrow or trabecular bone),
  - 1 mm/sec through dense cortical bone,
  - 0.5 mm/sec at locations of significant density change (cortical interfaces).

An enabling pedal allowed immediately stop and removal of the robot as required. Irrigation was performed using a Stryker CORE irrigation cassette and manual irrigation and suction following the cutting tip during resection.

**Fig. 3:**
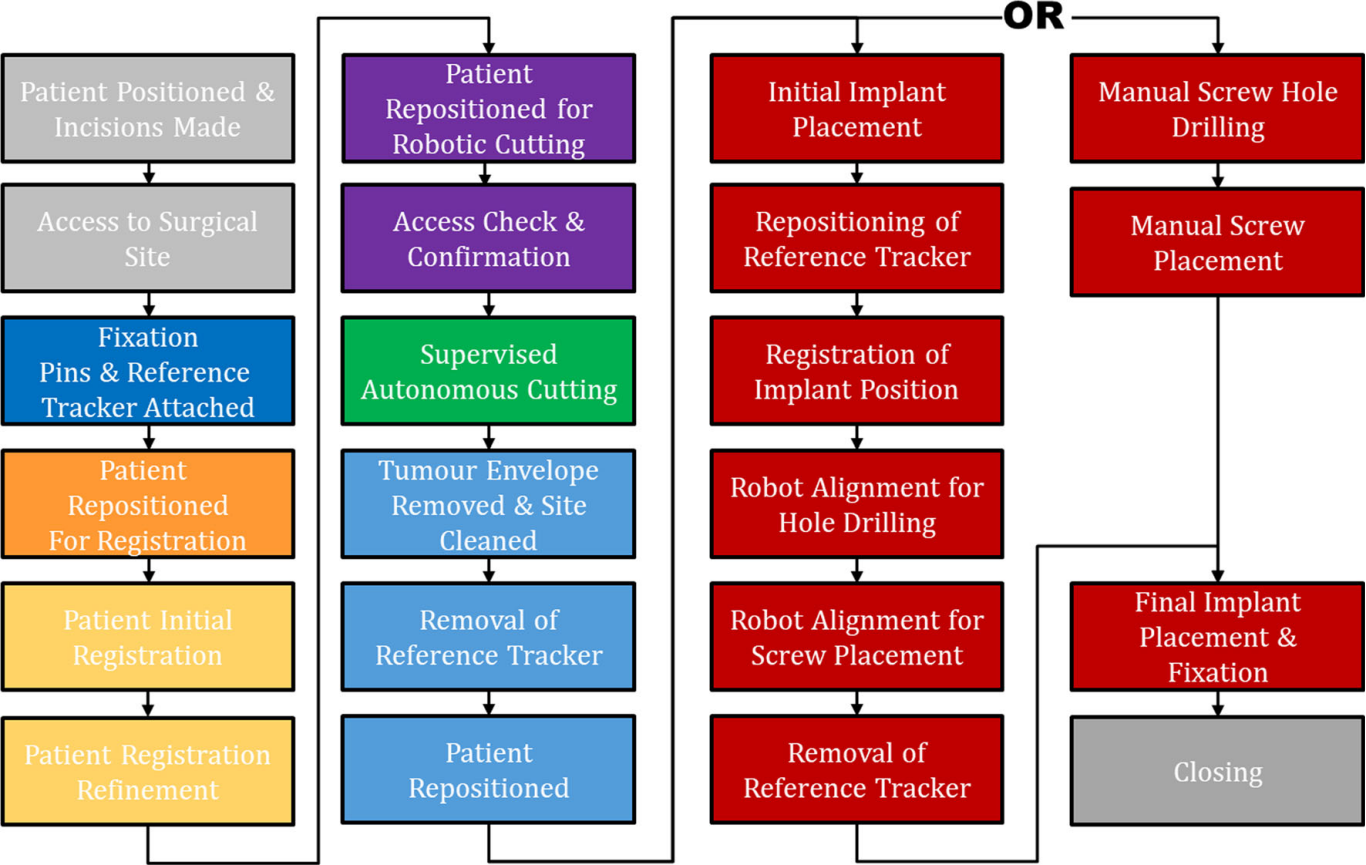
The overall intraoperative surgical workflow. The robotic screw drilling approach (third column) was used only for cases 3 & 4

After excision, the robot was removed from the surgical site, the leg detached from fixation and the sheep returned to lateral recumbency. All attached hardware was removed and the bone around the excised region cleaned of soft tissue. The patient-specific implant was placed into the cut and fixed with four screws: 2 × 3.5 mm diameter cortical screws proximally (AxSOS3 3.5-mm cortex screw, Stryker, USA), and 2 × 4.0 mm diameter trabecular screws distally (AxSOS3 4.0-mm cancellous screw, Stryker, USA). In 22 of the 24 cases, screw drilling and fixation was performed manually by the surgeon using a drill guide. In sheep 3 and 4, the robot was used to drill the holes before manual fixation. Robotic drilling was successfully performed; however, the additional repositioning steps extended surgical time and was not continued after these two initial cases. The surgical workflow is shown in Fig. 3.

After implant fixation, the surgical site was lavaged, photographed and the wound closed. Two post-operative orthogonal view radiographs were taken to assess implant and screw placement.

### Accuracy analysis approach

All subsequent analysis was performed on the first acquired post-operative CT scans as per Table 1: 8 weeks for the histology cohort, 12 weeks for the biomechanical cohort and immediately after euthanasia for posthumous sheep.

**Fig. 4:**
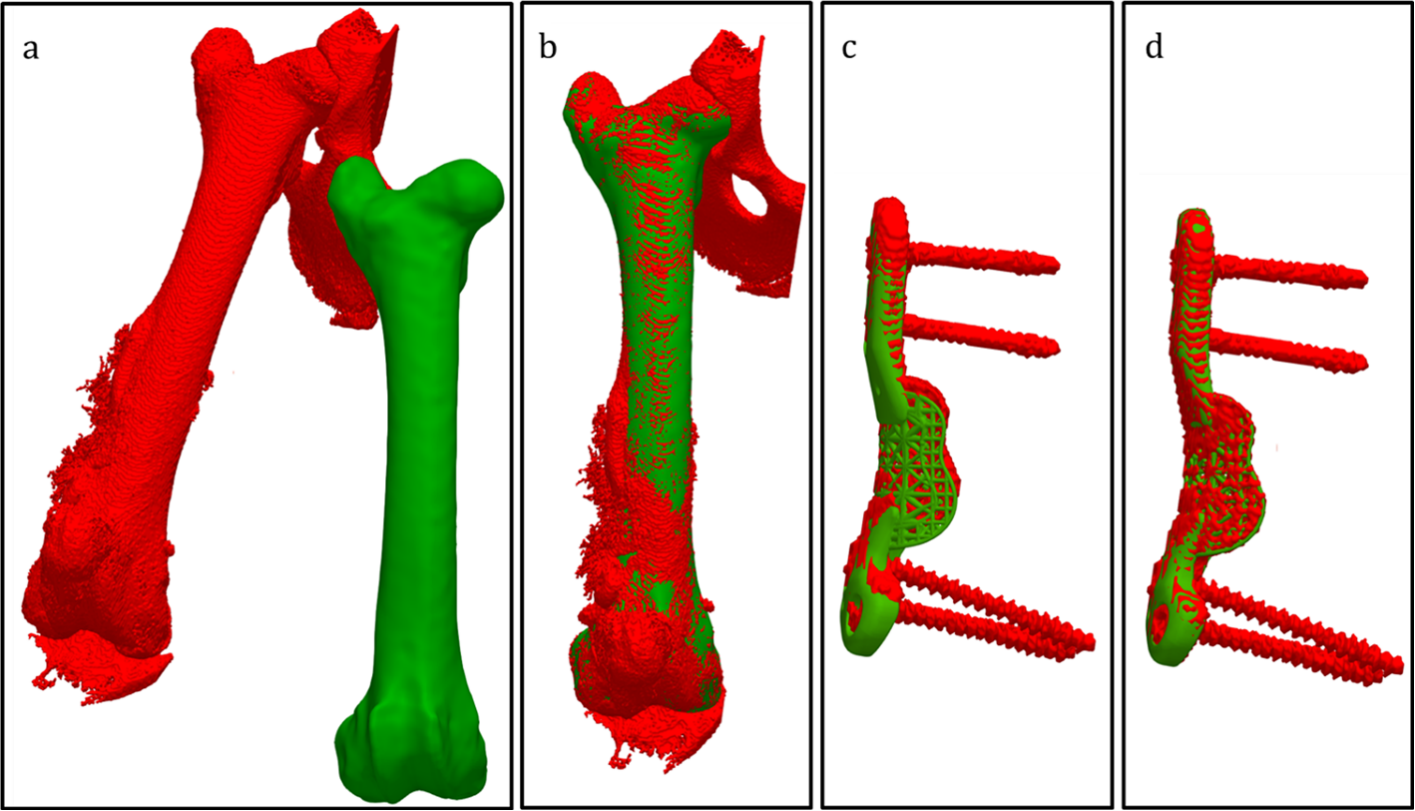
The multistep process for determining the implant placement error shown here for Sheep 9. Post-operative segmented data are shown in red; pre-operative plan data are shown in green. Post- and pre-operative image data are initially unaligned (**a**). A combination of manual manipulation and ICP of the segmented bone models is used for initial alignment (**b**). The error in implant placement (**c**) is the difference between the planned implant position (green) and segmented implant position after initial alignment (red). ICP is used to align the planned and segmented post-operative implant (**d**) with the resulting transformation representing the placement error (**d**)

Post-operative CT data were imported into the developed planning software and the right femur re-segmented. Post- and pre-operative bones were registered using ICP and the computed transformation applied to the post-operative image data, aligning it with the pre-operative plan. The actual position of the implant was extracted from aligned post-operative images using thresholding and registered to the planned implant model, providing the transformation between the planned and actual implant position. The process is shown visually in Fig. 4. Computed implant transformations were decomposed into individual rotation (ZYX Euler angles) and translation components in MATLAB to obtain implant placement error. The analysis coordinate system is shown in Fig. 1.

### Operative time analysis

Operative time was measured with a combination of intraoperative time tracking and post-operative video analysis. Opening and closing steps were excluded; operative workflow was divided as follows:

1. Fixation of pins and tracking hardware,
2. Sheep repositioning from lateral to dorsal recumbency,
3. Patient-to-image registration,
4. Repositioning and fixation of sheep to OR table,
5. Robotic cutting,
6. Repositioning of sheep from dorsal to lateral recumbency, removal of fixation hardware and cleaning of bone,
7. Drilling and tapping of screw holes and fixation of implant.

The first five steps were defined as “robotic” components and the last two as “implant” components related to the actual insertion and fixation of the implant. The four posthumous surgeries were excluded from time analysis.

## Results

**Fig. 5:**
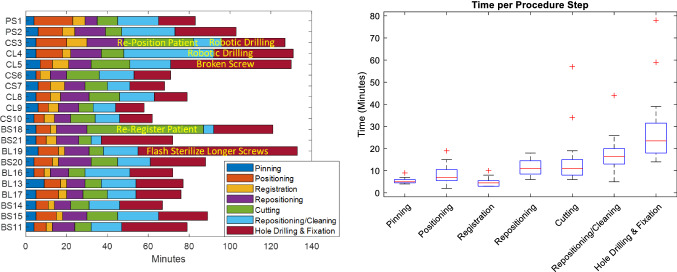
Time required for each case (left) and distributions of time required for each workflow step (right). The order of cases on the left represents the order in which surgeries were performed. Colours refer to equivalent steps in Fig. 3. Workflow challenges resulting in increased surgical time are indicated in text

**Fig. 6:**
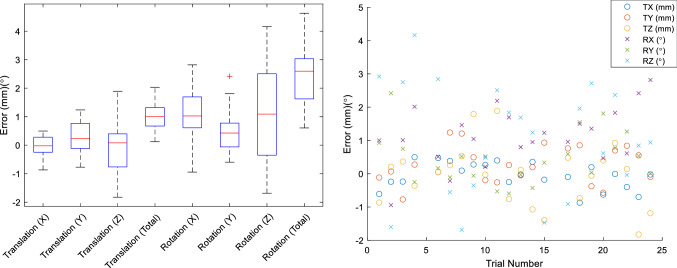
Distribution of observed implant positioning errors in translation and rotation (left) and implant positioning errors for each case (right)

Robotic cutting was successfully performed in all 24 cases. Three sheep were prematurely euthanized 4 h to 1 week after surgery due to fractures of the operative leg. In one case (Sheep 5) this was due to the breakage of one of the distal (trabecular) screws during fixation; fracture occurred 1.5 days after surgery. The cause of the second fracture (Sheep 10) is unknown and however is hypothesized to have been due to complications related to anaesthesia and load bearing immediately post-surgery. The third fracture (Sheep 16) occurred 1 week after surgery when the sheep slipped and fell on the operative leg while being moved between pens. Two fractures (Sheep 5 and 16) passed through the cut geometry, potentially resulting in shifts of the implant: these were excluded from analysis. The third fracture (Sheep 10) occurred above the level of the implant and was included in accuracy analysis. In total, 22 cases were used for implant position accuracy analysis; 20 cases were used for surgical time analysis. Overall surgical time and implant placement accuracy is shown Tables 2 and 3 and Figs. 5 and 6.

**Table 2:** Time required for surgical workflow components

Average time (minutes)						
Pinning	Positioning	Registration	Repositioning	Cutting	Repositioning/cleaning	Hole drilling/fixation
5.25 ± 1.21	8.35 ± 4.1	4.7 ± 1.95	11.3 ± 3.26	14.3 ± 11.82	16.9 ± 8.19	28.5 ± 15.62
43.9 ± 15.31					45.4 ± 19.97	
89.3 ± 25.25

**Table 3:** Implant placement errors. The analysis coordinate system is shown in Fig. 1

	Translation (mm)				Rotation (°)			
	*X*	*Y*	*Z*	Total	*X*	*Y*	*Z*	Total
Overall	− 0.06 ± 0.4	0.27 ± 0.56	− 0.05 ± 0.93	1.05 ± 0.53	1.13 ± 0.87	0.46 ± 0.78	1.05 ± 1.67	2.38 ± 0.98
Absolute	0.33 ± 0.23	0.49 ± 0.37	0.7 ± 0.6		1.23 ± 0.7	0.64 ± 0.63	1.66 ± 1.04	
RMS	0.39	0.61	0.91	1.17	1.31	0.89	1.94	2.56

A mean implant position error of 1.05 ± 0.53 mm was observed, in combination with a mean orientation error of 2.38 ± 0.98°

### Surgical time analysis

Recorded surgical time (excluding opening and closing) varied between 58 and 133 min, approximately evenly divided between robotic (43.9 ± 15.32) and implant-based (45.4 ± 18.97) tasks. A mean procedure time of 89.3 ± 25.25 min was observed.

### Implant positioning accuracy analysis

**Fig. 7:**
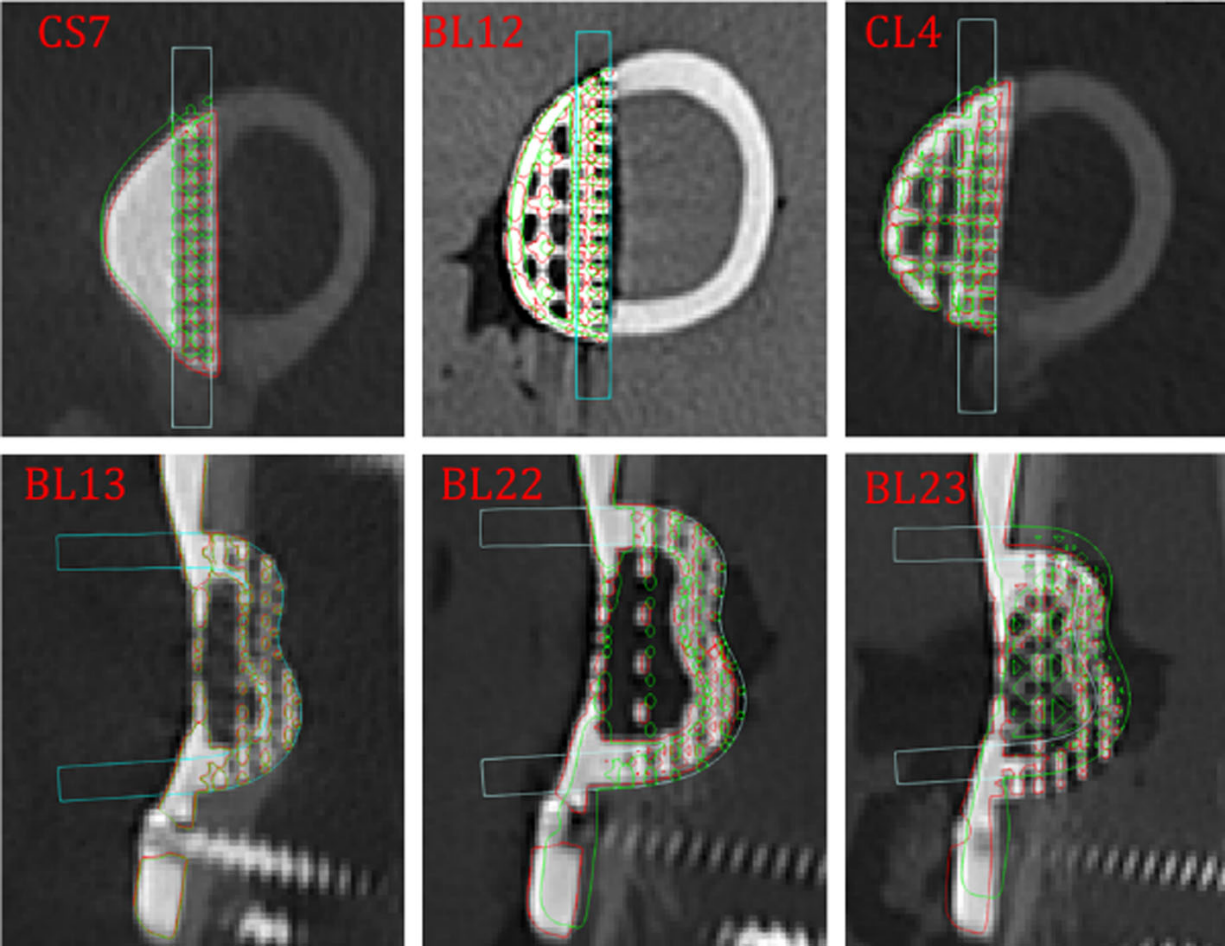
Minimum, median and maximum observed errors in the axial plane (top) and coronal plane (bottom). The segmented implant position is outlined in red, the planned implant position in green. The red text refers to the specific case ID

Examples of minimum, maximum and median orientation errors in the axial plane, and minimum, maximum and median position errors in the coronal plane are shown in Fig. 7.

**Fig. 8:**
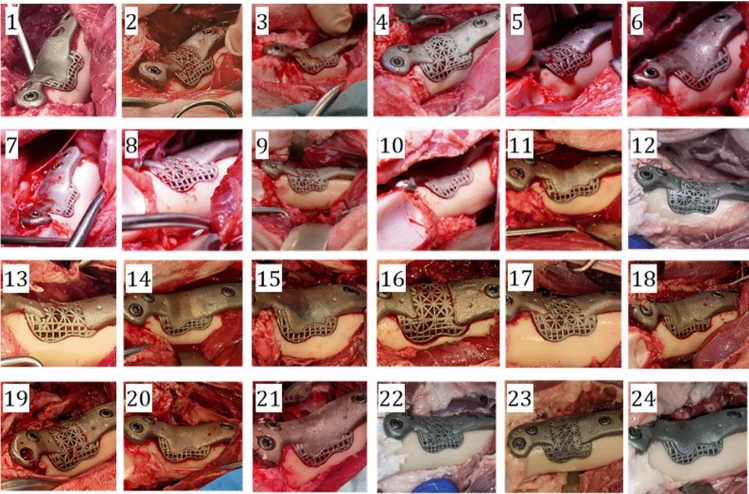
Intraoperative photographs of implant fit for all cases

Intraoperative photographs of all 24 implant positions are shown in Fig. 8. Cases 5 and 16 were excluded from analysis due to post-operative fracture. Surgery on sheep 12, 22, 23 and 24 was performed posthumously.

## Discussion

This work has described a system and workflow for robotic-assisted implantation of patient-specific orthopaedic implants. The approach was assessed as part of an animal trial examining the viability of additively manufactured patient-specific lattice implants. Previous evaluations of lattice structured implants in sheep have investigated segmental [27] or bone plug-type implants [28]. By comparison, this trial provides a realistic clinical model that includes the complete range of expected surgical errors and workflow factors. This work has demonstrated that high levels of implant placement accuracy can be achieved, with minimal gap between implant and remaining bone. Due to differences in analysis approach and anatomical factors, direct comparison of achieved accuracy levels with existing literature can be challenging. Bosma et al. [17] compared errors in cutting plane position on cadaveric femurs, achieving a best accuracy of 1.9 ± 1.1 mm when utilizing PSI. Wong et al. [18] compared the mean variation of a planar cut surface to the planned cut surface on the cadaver pelvis specimens, achieving a maximum accuracy of 1.37 mm when using PSI. Previous pilot tumour robotics work by Khan et al. [23] achieved a maximum deviation from the preoperative plan of 2.2 mm when using robotic assistance on anatomical phantoms. Some work also exists evaluating robotic assistance in placement of off-the-shelf implants, for example for total knee arthroplasty (TKA). Sires and Wilson [29] assessed the orientation error of implants placed using the MAKO robotic system for 29 patients, with absolute errors in femoral component positioning of 1.17 ± 1.1°, 1.79 ± 1.12° and 1.9 ± 1.88° in the coronal, sagittal and transverse planes (equivalent to the Y-, X-, Z-axes in this work), respectively.

The animal trial within which this work was completed was designed to also compare the bone ingrowth and biomechanical properties of lattice and solid patient-specific implants, when used in combination with surgical robotics. This introduced several limitations and compromises. As only one post-operative CT could be performed, the timing was defined allowing the maximum amount of information to be obtained about bone ingrowth and remodelling. In the intervening period the bones underwent observable post-operative remodelling, with differences in pre- and post-operative morphology potentially affecting the overall accuracy analysis. The same cut geometry was performed on all sheep: pre-trial evaluation demonstrated that the robot could accurately remove a range of complex geometries on technical and anatomical phantoms; however, this should be confirmed in more realistic settings. The sheep model introduced complications to the overall clinical workflow, which must be adjusted and confirmed in a human model. The direct fixation of the femur to the side rails of the OR table would likely not be suitable for a human patient; however, due to the short length of the femur alternative fixation methods (e.g. a leg holder or cast) were not appropriate. Bone fixation prevented a large amount of patient motion, with the robot compensating for remaining motion via real-time tracking of the patient and end-effector. Comparison of the biomechanical properties and remodelling characteristics of the implants is the subject of ongoing work and will be presented in subsequent publications.

Significant time variations were observed associated with initial learning curve and intraoperative complications. The extended time required in Sheep 3 and Sheep 4 was largely due to problems positioning the sheep to achieve adequate reachability and tracker visibility. Additional time was also taken when using the robot for drilling of screw holes due to further challenges with sheep positioning. One of the distal fixation screws broke within the bone during implant fixation for Sheep 5. The time taken attempting to remove and replace this screw significantly extended the surgical time. It was observed during Sheep 15 that no bone material was removed during the first two cutting passes, likely due to a shift in the patient tracker position after registration. The leg was re-registered and the cutting restarted without further incident. Sterilized screws of the required length were not available during the implant fixation process of Sheep 19: longer screws were flash sterilized using an onsite autoclave, extending the surgical time. Excluding cases with significant workflow issues and after learning curve (i.e. only the last 5 cases of the histology cohort and last 6 cases of the biomechanical cohort per Fig. 5) resulted in an overall mean surgical time of 73.83 ± 9.45 min (37.33 ± 5.85 min for robotic components, 36.5 ± 7.1 min for implant components).

The bulk of both position and orientation error occurred along and around the *z*-axis (see Fig. 1). Accurate registration of this direction was challenging as the bone is largely cylindrical and few constraining features were reachable: it was not possible to utilize cartilage-covered areas to avoid damage to the joint surface. There may also be a tendency for the surgeon to place the implant such that the gap at the top of the implant is minimized, with a larger gap at the bottom (which is difficult to see). Qualitatively, it was possible to achieve good fits between the implant and bone in all cases (Fig. 8). This demonstrates the ability for robotics to achieve repeatable and precise removal of complex bone volumes, and that these volumes can be precisely filled with additively manufactured patient-specific implants. It was not possible to accurately measure gap distances; however, initial assessment of post-mortem micro-CT has shown that distances were sufficiently small to enable bone ingrowth.

Future work will focus on further improvements to system accuracy through optimization of the registration process. New cutting tools are under development to allow deeper cuts and reduce kerf. More complex cutting geometries such as those described in [25] will also be investigated. Confirmation of the approach on human anatomy and direct comparison to free-hand cutting methods will also be the subject of future work.

## Conclusion

This work represents the first assessment of the combination of surgical robotics and patient-specific additively manufactured implants. A robotic system and workflow were developed and evaluated as part an animal trial on 24 healthy adult sheep, in which robotic bone excision was followed by placement of additively manufactured patient-specific implants. A mean overall implant position error of 1.05 ± 0.53 mm was observed, in combination with a mean orientation error of 2.38 ± 0.98°. Total recorded surgical time (from access to implantation, excluding opening and closing) varied between 58 and 133 min, with this approximately evenly divided between robotic (43.9 ± 15.32) and implant-based (45.4 ± 18.97) tasks. A mean procedure time of 89.3 ± 25.25 min was observed. The results demonstrate that the combination of robotics and patient-specific implants is feasible and that high levels of implant placement accuracy can be achieved, with minimal gap between implant and remaining bone.
